# Prefrontal cortex activity and functional organisation in dual-task ocular pursuit is affected by concurrent upper limb movement

**DOI:** 10.1038/s41598-024-57012-2

**Published:** 2024-05-01

**Authors:** Lénaïc Borot, Ruth Ogden, Simon J. Bennett

**Affiliations:** 1 School of Sport and Exercise Sciences, Faculty of Science, Liverpool John Moores University, Liverpool, UK; 2 School of Psychology, Faculty of Health, Liverpool John Moores University, Liverpool, UK

**Keywords:** Neuroscience, Cognitive neuroscience, Motor control

## Abstract

Tracking a moving object with the eyes seems like a simple task but involves areas of prefrontal cortex (PFC) associated with attention, working memory and prediction. Increasing the demand on these processes with secondary tasks can affect eye movements and/or perceptual judgments. This is particularly evident in chronic or acute neurological conditions such as Alzheimer’s disease or mild traumatic brain injury. Here, we combined near infrared spectroscopy and video-oculography to examine the effects of concurrent upper limb movement, which provides additional afference and efference that facilitates tracking of a moving object, in a novel dual-task pursuit protocol. We confirmed the expected effects on judgement accuracy in the primary and secondary tasks, as well as a reduction in eye velocity when the moving object was occluded. Although there was limited evidence of oculo-manual facilitation on behavioural measures, performing concurrent upper limb movement did result in lower activity in left medial PFC, as well as a change in PFC network organisation, which was shown by Graph analysis to be locally and globally more efficient. These findings extend upon previous work by showing how PFC is functionally organised to support eye-hand coordination when task demands more closely replicate daily activities.

## Introduction

Smooth pursuit and saccades are complementary but different functional outcomes of a similar cortico-ponto-cerebellar network^1,2^. Together, they ensure that gaze, and hence overt attention, is directed towards the object of interest, thus facilitating the processing of high acuity input from the central visual field, while at the same time enabling covert attention to process low acuity input from the peripheral visual field; for the spatial extent of covert attention during smooth pursuit see^3–5^. Importantly, smooth pursuit is not simply a reflexive response to retinal input^6,7^ and often involves cognitive processes such as attention, working memory and prediction^8^. Consequently, smooth pursuit may involve similar neural resources as secondary tasks presented at peripheral locations that require visual-spatial^9^ or colour^10,11^ working memory.

Specific areas of prefrontal cortex (PFC) are involved in the control of smooth pursuit, with activation varying between conditions where a moving object remains visible or is occluded. In the latter condition, participants exhibit a reduction in smooth pursuit velocity with the loss of retinal input^12^, followed by an anticipatory increase if the object reappears^13–15^. This is associated with increased activation of dorsolateral prefrontal cortex (DLPFC)^16^, which exhibits a negative correlation with the reduction in smooth pursuit velocity^17^. Findings of increased bilateral DLPFC activation during occlusion have also been reported^18^, although this was mediated by additional cues that influenced predictability of the occluded object trajectory. The authors suggested that activation of different areas of PFC during ocular pursuit depends on the requirement for higher-order cognitive processes. This is consistent with the areas of PFC (DLPFC, medial PFC—MPFC) being differentially activated by demands on attention, working memory and prediction^19–21^.

Here, we examined the impact of a secondary change-detection task (visual-spatial or colour working memory) embedded within a spatial prediction motion task, on DLPFC and MPFC measured using functional Near InfraRed Spectroscopy (fNIRS). These two areas of PFC have been implicated in working memory and related executive functions^22^, and are involved in pursuit tasks^17,18^. Consistent with previous studies on prediction motion^23^, we expected participants to exhibit a decrease in judgment accuracy when the object reappeared close but behind the correct location. For the secondary change-detection task, we expected a decrease in judgement accuracy as a function of demand on working memory. Moreover, we expected that the increased demand on working memory in the change-detection task would result in changes in PFC activity and organisation.

Extending previous imaging work described above, we required participants to pursue the moving object of the prediction motion task with eyes alone, or eyes and upper limb. Afferent and efferent signals from the upper limb have been shown to facilitate smooth pursuit^24^, even when the moving object undergoes an occlusion^25,26^. Modelling of behavioural data indicates a sharing of afferent and efferent signals between the oculomotor and motor systems, which act interdependently to achieve the task goal^27^. Accordingly, we expected that smooth pursuit would benefit from concurrent and congruent upper limb movement. It is less clear, however, whether afferent and efferent signals from the upper limb would facilitate prediction motion^28^ and change-detection judgment accuracy. Investigating whether upper limb tracking mediates the demand on attention and working memory, and how this affects PFC activity and efficiency of organisation, could help in understanding cortical control of pursuit tasks that are representative of everyday interactions.

## Results

Given the novelty of our protocol, it was first necessary to determine if participants performed the dual-task pursuit as expected. To this end, we examined the effect of Position Step (*−4, −2, *+*2, *+*4 deg*), Stimulus Array (*Control, Colour, Form*) and Tracking (*OC, OM*) on behavioural measures (judgment accuracy; response time) from the prediction motion and change-detection tasks. For smooth pursuit of the moving object in the prediction motion task, we included an additional fixed effect of Time (*T1, T2, T3*) to determine the impact of removing visual feedback of the moving object during occlusion. Next, we evaluated the working memory demands of the change-detection task on PFC activity and organisation, and whether this was mediated by afferent and efferent signals from concurrent upper limb movement. Given the equivocality regarding activation of left and/or right DLPFC in pursuit tasks, and the lack of research on MPFC, our exploratory analysis for mean O_2_Hb and HHb in each ROI, as well as global efficiency, investigated the effect of Tracking (*OC, OM*) and Stimulus Array (*Control, Colour, Form*). For local efficiency, we also included an additional fixed effect of Channel.

### Behavioural measures

#### Prediction motion

**Figure 1:**
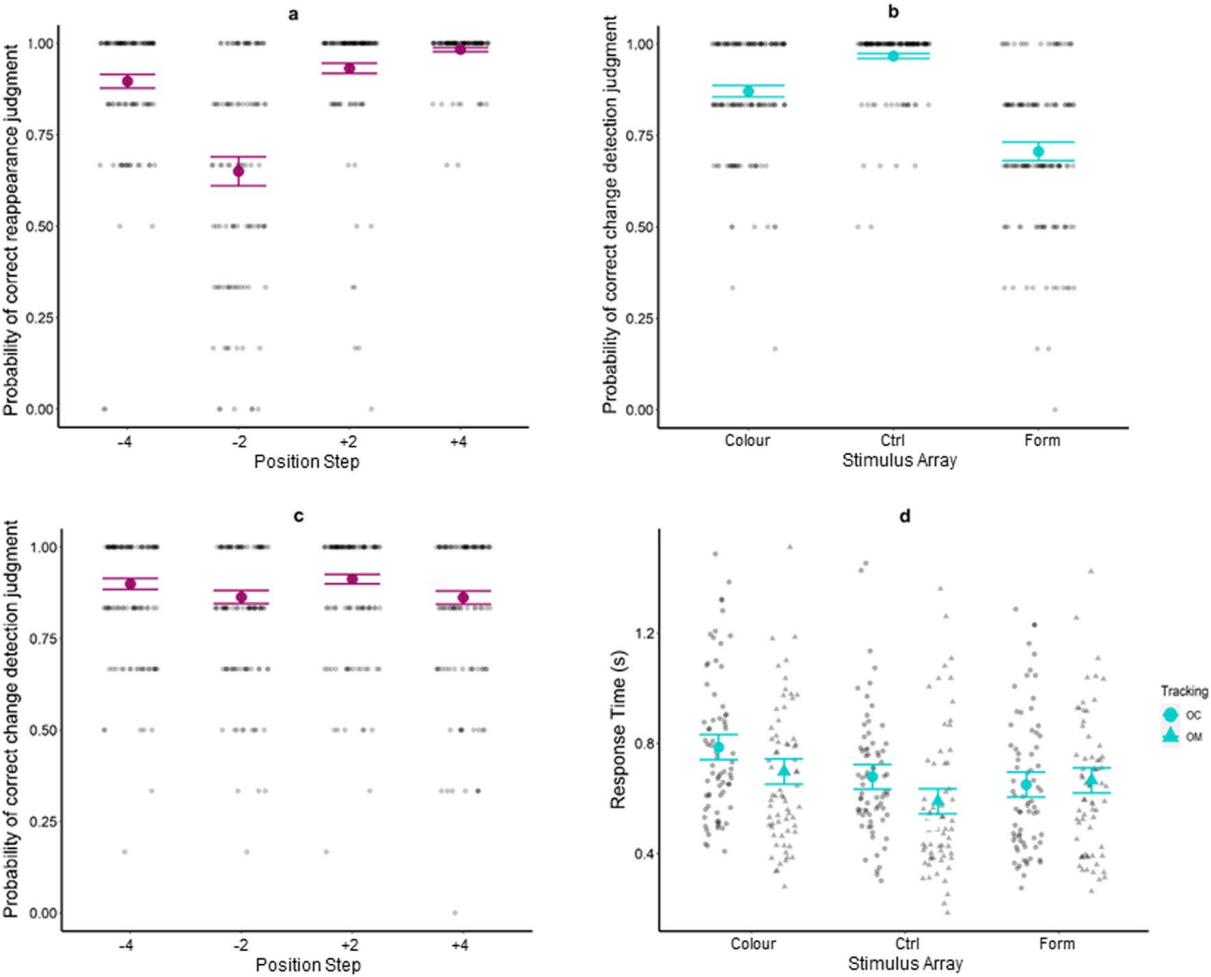
Probability of correct judgment for each combination of Position Step (−4, −2, +2, +4 deg) in the prediction motion task (**a**). Probability of correct judgment in the change-detection task for each combination of Stimulus Array (Colour, Control and Form; **b**). Probability of correct judgment for each combination of Position Step (−4, −2, +2, +4 deg) in the change-detection task (**c**). Response time in the change-detection task for each combination of Stimulus Array and Tracking (**d**). Estimated marginal means (large, coloured markers) and the standard errors are shown from the accepted model. For (**a**)–(**c**), individual data are represented as small, high-transparency dots and correspond to the individual probability of a correct judgement for each level of the factors not represented on the x-axis. For (**d**), individual data are represented as small, high-transparency dots and correspond to the average response for each level of the factors not represented on the x-axis. For all panels, a small horizontal jitter has been introduced in order to reduce the overlap between individual data.

For judgement accuracy the reduced model (AIC = 1014.20; marginal R^2^ = 0.61; conditional R^2^ = 0.78) indicated a significant main effect of Step [χ^2^ (3) = 256.97; p < 0.001]. Consistent with our hypothesis, it can be seen in Fig. 1a that judgments were less accurate (p < 0.0001) when the object reappeared with a small negative step (− 2 deg: 0.65) compared to all other steps (+ 2 deg: 0.93, OR = 7.38; + 4 deg: 0.98, OR = 31.14; − 4 deg: 0.90, OR = 4.64). Judgments were also more accurate (p < 0.0001) when the object reappeared with a large positive step compared to small positive step (OR = 4.22) and large negative step (OR = 6.71).

For eye velocity, the reduced model (AIC = 1765.90; marginal R^2^ = 0.35; conditional R^2^ = 0.66) had significant main effects of Stimulus Array [χ^2^ (2) = 18.57; p = 0.001], Tracking [χ^2^ (1) = 14.42; p = 0.001] and Time [χ^2^ (2) = 1195.85; p = 0.001], as well as a significant Stimulus Array x Tracking interaction [χ^2^ (2) = 6.80; p = 0.033]. As expected, eye velocity was highest with vision of the moving object just prior to occlusion (2.76 deg/s), decreased at onset of occlusion (2.21 deg/s, MD = 0.55), and then decreased further as the occlusion interval progressed (1.59 deg/s, MD = 0.62). Decomposition of the significant interaction effect revealed that eye velocity was significantly (p = 0.0003, MD = 0.20) higher in the *OM* (2.32 deg/s) than *OC* (2.12 deg/s) tracking condition for the *Form* stimulus array. There was no difference in eye velocity between the *OM* (2.14 deg/s, 2.25 deg/s) and *OC* (2.07 deg/s, 2.22 deg/s) tracking conditions for the *Colour* and *Control* stimulus arrays, respectively. In the *OC* tracking condition, eye velocity was significantly higher in the *Control* than *Colour* stimulus array (p = 0.02, MD = 0.15). In the *OM* tracking condition, eye velocity was significantly higher in the *Form* than *Colour* stimulus array (p = 0.002, MD = 0.18).

#### Change-detection

For judgment accuracy, the reduced model (AIC = 944.56; marginal R^2^ = 0.61; conditional R^2^ = 0.70) indicated a significant main effect of Stimulus Array [χ^2^ (2) = 181.36; p < 0.001]. As expected, judgment accuracy for the *Control* stimulus array (0.97) was higher (p < 0.0001) than the *Form* (0.71, OR = 12.20) and *Colour* stimulus arrays (0.87, OR = 4.34). As shown in Fig. 1c, judgment accuracy was lower for the *Form* than *Colour* stimulus array (p < 0.0001, OR = 2.81). There was also a main effect of Step [χ^2^ (3) = 14.01; p = 0.003] but no interaction with Stimulus Array. As shown in Fig. 1b, judgment accuracy on the change-detection task was higher for trials in which the primary pursuit object reappeared with a small positive position step (+ 2 deg: 0.91) than a small negative position step (− 2 deg: 0.86, OR = 1.65, p = 0.017) or a large positive position step (+ 4 deg: 0.86, OR = 1.66, p = 0.013).

For response time, the reduced model (AIC = − 121.97; marginal R^2^ = 0.05; conditional R^2^ = 0.45) indicated a significant main effect of Stimulus Array [χ^2^ (2) = 23.99; p < 0.001] and Tracking [χ^2^ (1) = 7.86; p = 0.005], as well as a significant Stimulus Array x Tracking interaction [χ^2^ (2) = 6.63; p = 0.04]. Response time in the *OC* tracking condition was longer for the *Colour* (0.786 s) than *Control* (0.678 s, MD = 0.11, p = 0.015) and *Form* (0.650 s, MD = 0.14, p = 0.0005) stimulus arrays. In the *OM* tracking condition, response time was longer for the *Colour* (0.698 s) than *Control* (0.590 s, MD = 0.11, p = 0.02) stimulus array (Fig. 1d).

### Neuroimaging measures

#### Activity

**Figure 2:**
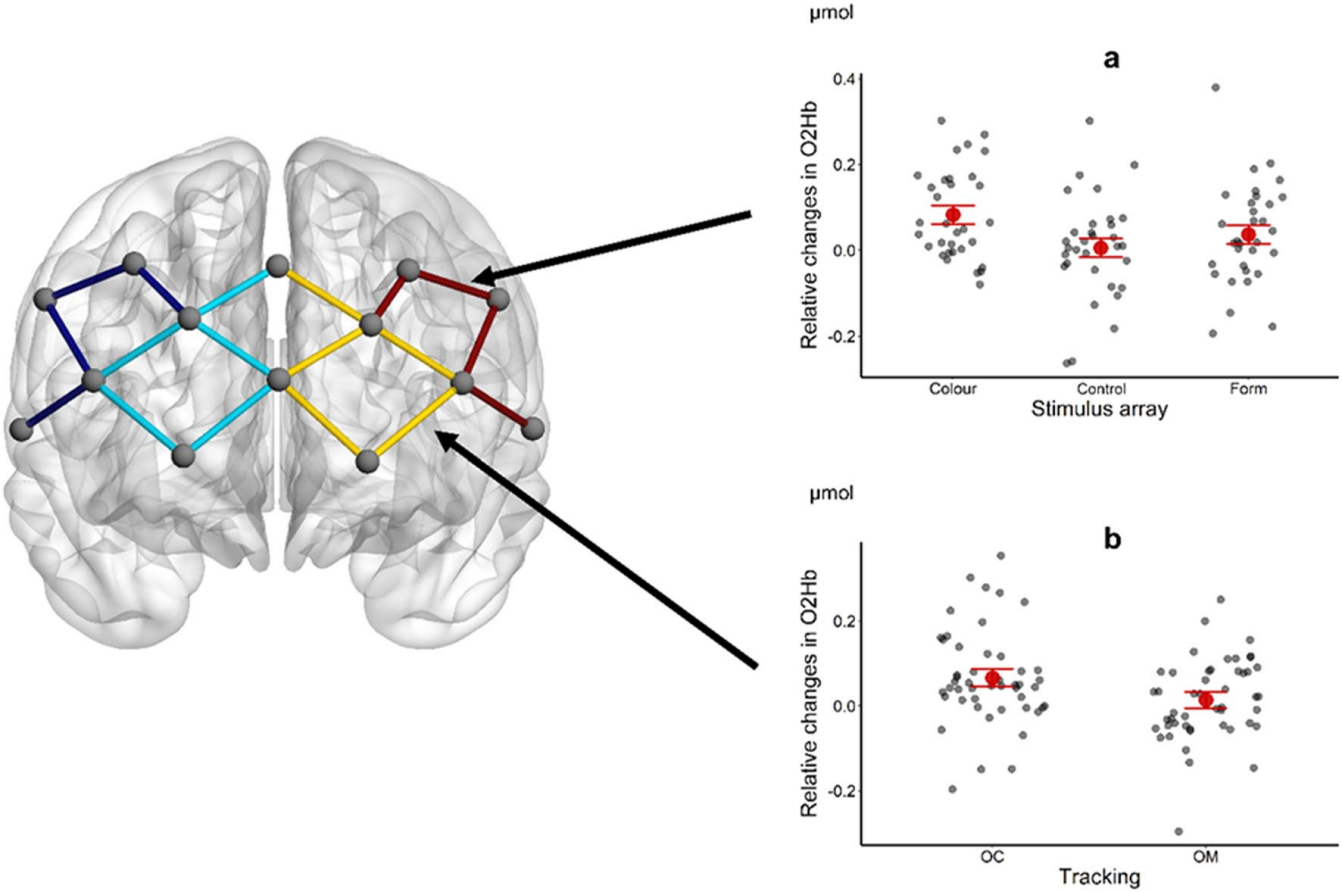
Representation of the channels involved in each ROI (left), with the latter in different colours: dark blue = right DLPFC; light blue = right MPFC; yellow = left MPFC; red = left DLPFC. Mean O_2_Hb in left DLPFC as a function of Stimulus Array (**a**) and left MPFC as a function of Tracking Condition (**b**). Estimated marginal means (large, coloured circles) and the standard errors are shown from the accepted model. Individual data are represented as small, high-transparency dots and correspond to the average response for each level of the factors not represented on the x-axis. For all panels, a small horizontal jitter has been introduced in order to reduce the overlap between individual data.

For left DLPFC (Fig. 2b), the reduced model (AIC = − 141.4; marginal R^2^ = 0.07; conditional R^2^ = 0.23) indicated a significant main effect of Stimulus Array [χ^2^ (2) = 8.85; p = 0.012]. Consistent with our expectation of an increased demand on working memory, mean O_2_Hb was higher for *Colour* stimulus array (0.08 µmol, MD = 0.08, p = 0.012) than the *Control* stimulus array (0.006 µmol). Mean O_2_Hb for the *Form* stimulus array (0.04 µmol) was intermediate between the other two stimulus arrays (Fig. 2b). However, there was no such effect for right DLPFC, with the full model (AIC = − 176.68; marginal R^2^ = 0.04; conditional R^2^ = 0.43) providing no better fit than the intercept-only model (AIC = − 180.13; conditional R^2^ = 0.41).

For left MPFC (Fig. 2a) the reduced model (AIC = − 175.74; marginal R^2^ = 0.06; conditional R^2^ = 0.41) indicated a significant main effect of Tracking [χ^2^ (1) = 9.61; p = 0.0019]. As shown in Fig. 2a, mean O_2_Hb was greater in the *OC* (0.07 µmol) than *OM* tracking condition (0.01 µmol, MD = 0.05). For right MPFC, the full model (AIC = − 138.77; marginal R^2^ = 0.03; conditional R^2^ = 0.38) indicated no significant main or interaction effects and was rejected in favour of the intercept-only model (AIC = − 143.46; conditional R^2^ = 0.35).

For Mean HHb, no significant main or interaction effects were found, leaving us to accept the intercept-only model for left DLPFC (AIC = − 486.27; conditional R^2^ = 0.07), right DLPFC (AIC = − 355.22; conditional R^2^ = 0.16), left MPFC (AIC = − 396.96; conditional R^2^ = 0.05) and right MPFC (AIC = − 344.54; conditional R^2^ = 0.15).

#### Network organisation

**Figure 3:**
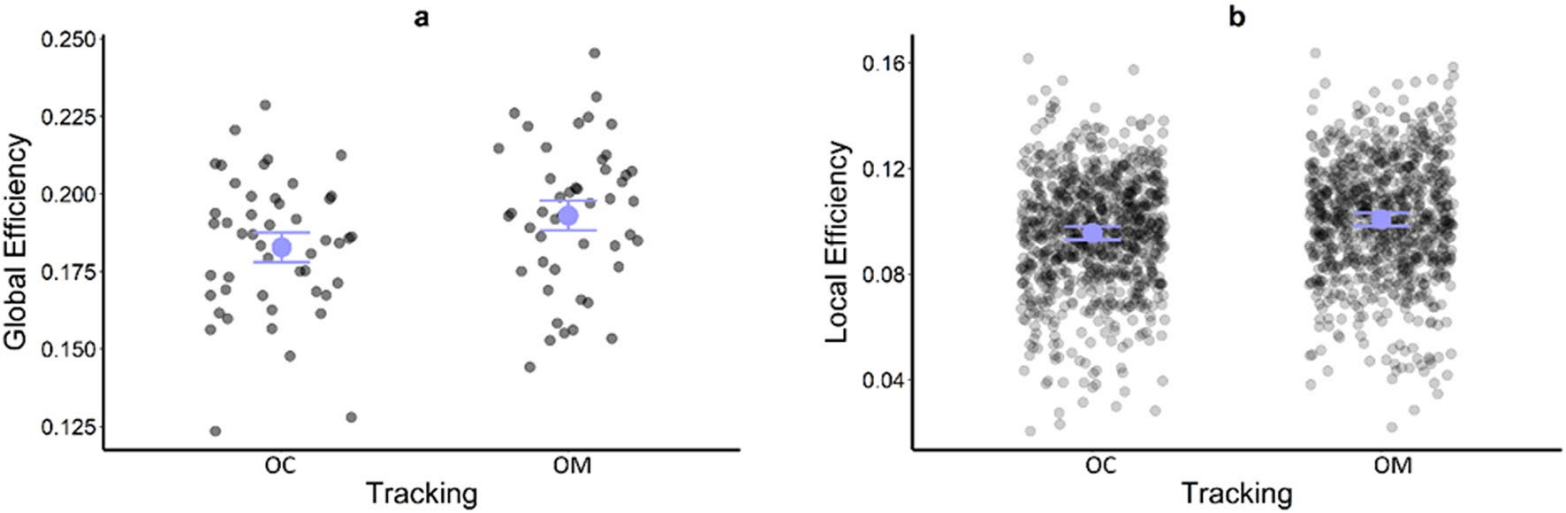
Global efficiency (**a**) and Local efficiency (**b**) as a function of Tracking Condition, with estimated marginal means (large, coloured circles) and the standard error from the accepted model. Individual-participant data are represented by small, high-transparency dots and correspond to the average response for each level of the factors not represented on the x-axis. For all panels, a small horizontal jitter has been introduced in order to reduce the overlap between individual data.

The reduced model (AIC = − 486.27; marginal R^2^ = 0.05; conditional R^2^ = 0.58) for global efficiency indicated a main effect of Tracking [χ^2^ (1) = 10.79; p = 0.001]. As shown in Fig. 3a, global efficiency was higher in the *OM* (0.19) than *OC* tracking condition (0.18, MD = 0.01).

For local efficiency, the reduced model (AIC = − 8687.3; marginal R^2^ = 0.09; conditional R^2^ = 0.28) had a significant main effect of Tracking [χ^2^ (1) = 32.89; p < 0.001] and Channel [χ^2^ (17) = 169.50, p < 0.001]. As can be seen in Fig. 3b, local efficiency was higher in the *OM* (0.101) than the *OC* tracking condition (0.096, MD = 0.005). Bonferroni-corrected pairwise comparisons indicated local efficiency differed between many of the channels, but a clear pattern was for higher local efficiency in channels located with left and right MPFC (for pairwise differences see Supplementary Material).

## Discussion

Increased demands on cognitive processes such as attention, working memory and prediction during ocular pursuit of occluded objects results in greater PFC activity^16–18^. Extending these previous studies, we designed a novel dual-task pursuit protocol to determine the effects on PFC (DLPFC and MPFC) of a secondary change-detection task (visual-spatial or colour working memory), embedded within a prediction motion task. Participants performed the primary task with eyes alone or eyes and upper limb (i.e., arm), thus enabling us to determine the contribution of extra-retinal (afference and efference) signals from concurrent upper limb movement, which have been shown to enhance smooth pursuit of an occluded moving object^25,26^.

Consistent with previous work on spatial prediction motion^23^, judgments were less accurate when the pursuit object reappeared behind the correct location with a small negative position step (− 2 deg). The suggestion is that participants tend to underestimate object location along the occluded trajectory^29,30^, resulting in gaze being closely aligned with the object reappearance, and thus making judgments more difficult^23,28^. In addition, there was no effect of the change-detection stimulus array on judgments of reappearance location, indicating that the allocation of attentional and working memory resource to the secondary task did not impair performance of the primary task. As expected, change-detection accuracy was highest for the control stimulus array. However, participants were less accurate at detecting a change in the form than the colour of the stimulus array. The lower accuracy was not associated with an increased response time, which was in fact longest for the colour stimulus array. This may be indicative of a speed-accuracy relationship whereby participants achieved a high level of accuracy for the colour stimulus array by taking longer to give a response. Finally, there was no difference in judgment accuracy of the primary and secondary tasks between ocular and oculo-manual tracking conditions. The implication is that although extra-retinal signals from upper limb movement may impact upon smooth pursuit eye movements^26^, they do not necessarily affect the judgement of object reappearance^28,31^. Indeed, we found the expected reduction in eye velocity following the loss of visual feedback, which continued as the occlusion interval progressed^12,13^, as well as evidence of a facilitatory effect from upper limb extra-retinal signals when the change-detection task involved the form stimulus array^25,26^.

The facilitatory effect of oculo-manual tracking on smooth eye movement was less prevalent than originally expected, even though participants were instructed to match the amplitude of object and upper limb motion, and given an opportunity to familiarise with the task. Previous work has shown that facilitation of smooth pursuit is greatest when the object motion is internally generated, cyclical with a duration of several seconds, and involves large amplitude upper limb movement^25^. Our use of discrete, short duration, externally-generated object motion may have limited the sharing of information between ocular and motor control centres, and thus the facilitatory effect. It might also be suggested that this could also have been influenced by not having visual feedback regarding the ongoing hand movement. However, oculo-manual facilitation was found in previous work, where vision of an object attached to the moving limb was initially available and then removed for several cycles of limb motion^25^. Importantly, oculo-manual facilitation was also observed in a condition where vision of the limb and an externally-generated object motion was not available throughout^32^. In the latter study, there was evidence of a training effect on smooth pursuit eye movement after several minutes in adults, which was not simply a result of improved accuracy of upper limb tracking. Whether such a training effect with the current protocol would have influenced smooth eye movement, and thus performance of the primary and/or secondary tasks remains to be seen. According to the scheme proposed in previous research^28^, the estimated displacement of an occluded moving object depends on a comparison between predicted and actual reappearance location (internalized), as well as current eye and actual reappearance location (externalized). Interestingly, however, the weight given to the externalized cue was reduced in oculo-manual pursuit of internally-generated object motion, implying that any training effect may depend on an improved prediction of the occluded object trajectory within the oculomotor system. If this can be trained^33^, an improved trajectory prediction in the primary task might aid judgments of reappearance location, and potentially free-up attentional and working memory resource for detecting changes in form or colour of the stimulus array.

Our analysis of cortical activity and network organisation sought to determine if there were any changes as a function of the secondary change-detection task, and whether this differed between the ocular and oculo-manual pursuit conditions. An effect of the secondary change-detection task on mean O_2_Hb was primarily found in left DLPFC. As could be expected given the role of DLPFC in working memory^34,35^, mean O_2_Hb was lowest with the control stimulus array where participants knew in advance that there would be no change between cue and probe. Conversely, changes in mean O_2_Hb were highest when participants were required to detect a colour change in the stimulus array. As described in the preceding section, participants were better at detecting a change in colour than form of the stimulus array, but it took them longer to give their response. As a subsidiary analysis, we investigated whether higher mean O_2_Hb in left DLPFC was related to participants spending more time responding to the colour stimulus array. The model (AIC = −132.65; marginal R^2^ = 0.075; conditional R^2^ = 0.24) indicated a significant main effect of Stimulus Array [χ^2^ (2) = 7.76; p < 0.03], but no significant effect of the covariate response time [χ^2^ (1) = 0.09; p > 0.05]. It would seem, therefore, that response time per se did not impact upon the change in mean O_2_Hb, and instead that it was related to processing activities that occurred when faced with the colour stimulus array.

Although there was no systematic effect of the secondary change-detection task on activity in MPFC, we did find evidence of an effect for tracking condition. This was most obvious in left MPFC, with a lower mean O_2_Hb in the oculo-manual than ocular tracking condition. Extending the behavioural findings discussed above, these data could indicate that extra-retinal signals from the upper limb do exert some influence on the attentional and working memory processes involved in dual-task pursuit, thereby reducing the cost for MPFC. In fact, while DLPFC is typically cited as a key area for working memory processes, such as those involved in representing an occluded object trajectory^17,18^, MPFC is involved in many cognitive processes^22,36^, and monitors other areas of PFC^37^. Of relevance to the current study is the role of MPFC in “cognitive branching”^38^, which refers to situations requiring the maintenance/monitoring of a primary task goal while simultaneously allocating attention to a secondary task goal^39^. In the dual-task pursuit protocol, it is feasible that extra-retinal signals from the upper limb influenced the need for ongoing monitoring of the primary prediction motion task, and thus the associated processing demand in MPFC.

The influence of upper limb tracking in the dual-task pursuit protocol of the current study was found to extend beyond individual ROIs. At both a local and global level, network organisation in PFC was more efficient in the oculo-manual than ocular tracking conditions. This is consistent with a network organisation that supports simultaneous integration and segregation of brain function^40^, which would presumably be beneficial when there are several concurrent sources of information to process and tasks to complete. Nonetheless, this PFC organisation did not appear to be associated with increased judgment accuracy of the primary or secondary tasks, which did not differ between ocular and oculo-manual tracking conditions. That said, oculo-manual tracking did not simply direct attention away from the primary task or act as a further task that competed for processes involved in the primary and/or secondary task. From a behavioural perspective, such an effect has recently been shown in a similar task requiring visual-spatial motion prediction^41^. A key difference compared to the current study is that here the upper limb was used to pursue the moving object, whereas in previous studies^41^ the upper limb was used to respond to a secondary interceptive timing task. The authors suggested that the condition with an upper limb movement resulted in two concurrent temporal estimations being monitored/performed, which placed an additional demand on processes occurring within the same cortical-subcortical network.

Here, it should be mentioned that although we found O_2_Hb in PFC changed as a function of the demands of our dual-task protocol, there was no evidence of a parallel change in HHb. We do not have a definitive answer for why this theoretical patten in the two chromophores was not observed in our fNIRS data, but it could in part be related to the fact that changes in O_2_Hb are usually of higher amplitude than changes in HHb^42^. It is also important to note that we included several control measures such as a baseline comparison condition, short-distance channels, covering channels with a piece of black material to minimize cross-talk from Eyelink IR illuminator, and preprocessing steps to improve signal quality (see^43^). Therefore, we contend that our results are more likely to represent task-evoked changes in the hemodynamic response than a false positive as consequence of a confounding factor. That said, it should be recognized that a two-stage procedure was applied for the control of Type 1 errors in the current study. At the first stage, reduced models for each dependent measure (n = 14) were derived using an iterative, top-down process in which main and interaction effects were retained at p < 0.05. At the second stage, Bonferroni-corrected pairwise comparisons were performed, thus maintaining p < 0.05 for the decomposition of each significant main and/or interaction effect. Therefore, given that the number of statistical tests performed across the 2 stages, it is likely that at least one of the significant effects was a false positive.

## Conclusion

We showed that activity and organisation of PFC was influenced by the increased demands on attentional and working-memory processes of performing a secondary change-detection task embedded within a prediction motion task. This was mediated by performing concurrent upper limb movement, and hence the availability of extra-retinal input. Future study is required to further characterise the hemodynamic (O_2_Hb and HHb) response in dual-task protocols, potentially including additional dependent measures (e.g., area under curve, peak concentration, time to peak concentration, slope fitted to curve), and/or neurophysiological measurements (e.g., EEG, MEG) that provide more direct assessment of cortical activity with higher temporal resolution. This could also consider the wider brain network, such as the fronto-parietal network that controls eye-hand coordination^44^. Indeed, although there is some recent work on functional connectivity between visual, parietal and frontal areas during smooth pursuit^45,46^, the influence of higher cognitive control or the need to perform concurrent tasks remains to be determined. Tasks with competing demands are commonplace in normal daily settings, and are sensitive to changes in cognitive function associated with acute and chronic neurological conditions^47^.

## Materials and methods

### Participants

Nineteen participants (10 males, 9 females) with a mean age of 26.9 (± 4.6) years from the staff and student population of the host University took part in the study. All participants were right-handed and self-declared with normal or corrected vision and no neurological impairment. Participants provided written informed consent prior to participation in the study. The study received ethics clearance through the Liverpool John Moores University Research Ethics Committee (20/SPS/014), and was conducted in accordance with the ethical standards specified by the Declaration of Helsinki 2008.

### Task and procedure

Participants were invited to come to the laboratory to carry out a test session of about two hours. They were seated on a height-adjustable chair at a worktop, such that their eyes were 915 mm away from a 24-inch LCD screen (ViewPixx EEG) operating at a resolution of 1280 × 1024 pixels and 100 Hz refresh rate. The head was supported by a chin rest in order to minimize head movement (during blocks of experimental trials). An EyeLink 1000 (250 Hz sampling rate) with remote optics was located beneath the lower edge of the LCD screen. Participants’ gaze location was calibrated relative to the LCD screen using a 9-point grid. The task was verbally explained to participants, and they were given the opportunity to familiarise with the protocol by performing 8 randomly-selected trials in both the ocular and oculo-manual tracking conditions before commencing the experimental phase of the study.

**Figure 4:**
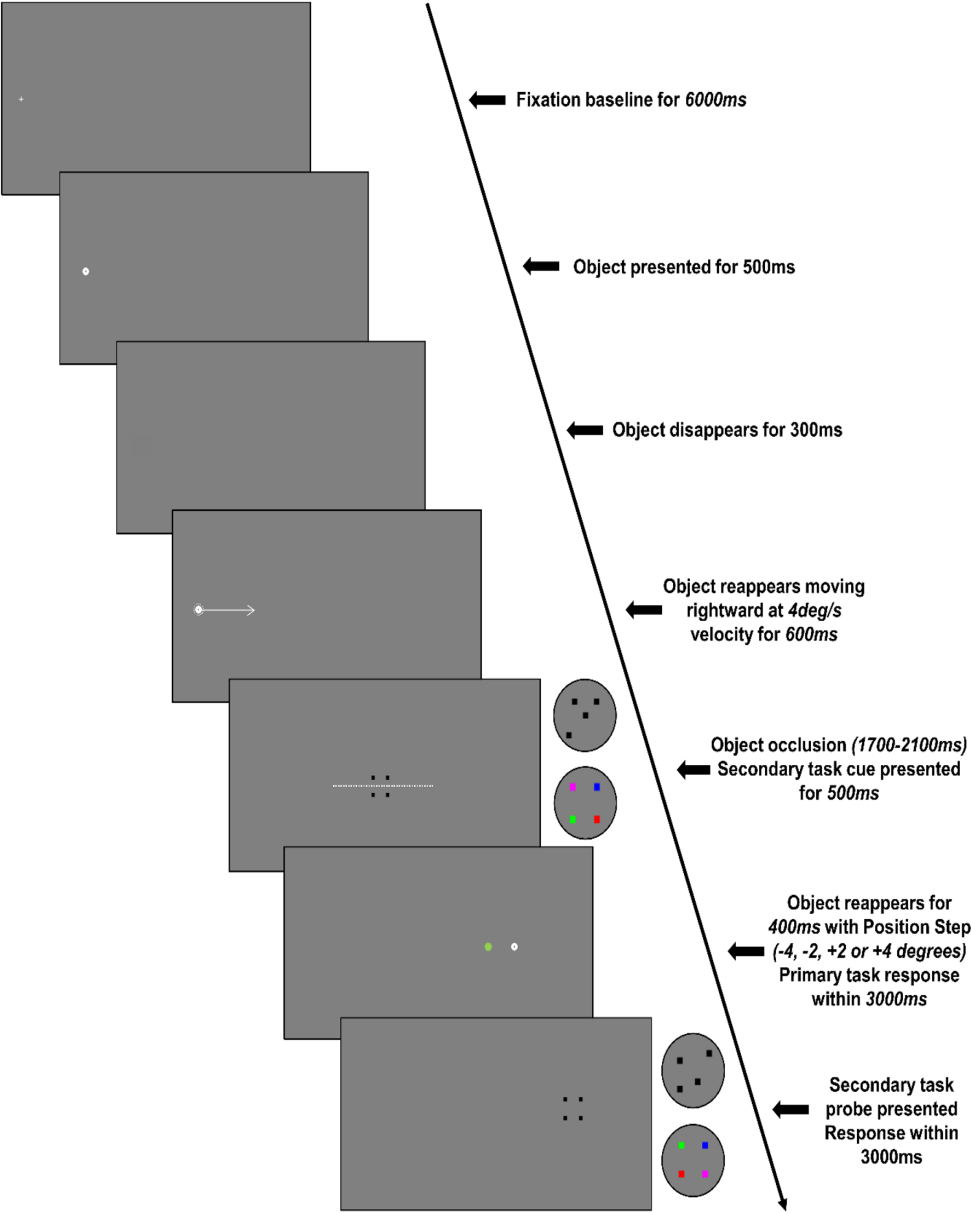
Schematic diagram showing the timeline of a trial for the control stimulus array (enlarged examples of a form and colour stimulus array are shown within the circle to the right of the grey boxes). Nb. White arrow depicting direction of object motion and white broken line depicting occluded object trajectory were not visible to participants.

Participants performed a novel, dual-task pursuit protocol that placed specific demands on visual-spatial and colour working memory (Fig. 4). The stimulus was generated using the Cogent toolbox v1.33 in Matlab® (MATLAB R2013b, The MathWorks, USA). Each trial started with 6000 ms fixation, where a cross was displayed at − 8.5 degrees to the left of screen centre (grey background). This coincided with the start location of object motion and ensured that participants did not have to relocate the eye before the start of each trial. The fixation cross then changed to a white circular object (0.5 degrees diameter) with a black dot at its centre. After 500 ms, the object disappeared for 300 ms and then reappeared moving horizontally to the right with a constant velocity of 4 deg/s. After 600 ms, the moving object disappeared for a random duration of 1700, 1800, 1900, 2000 or 2100 ms, and then reappeared for a further 400 ms. Importantly, the moving object reappeared on each trial with a position step that was −4, −2, +2 or +4 degrees from the correct position had the object continued to move with constant velocity. Participants were informed that their primary task was to judge whether the moving object reappeared behind or ahead of the correct location (i.e., prediction motion). This judgement had to be made within a 3000 ms interval after the moving object reappeared and required participants to press the z (behind) or v (ahead) key of the computer keyboard with their left hand. During each trial, participants pursued the moving object with either the eyes alone (ocular condition, *OC*) or with the eyes and right upper limb (oculo-manual condition, *OM*). For oculo-manual pursuit, movement of a stylus held in the right hand was measured with a Wacom A3 wide digitising tablet (250 Hz), located between the participant and the LCD screen. The recorded x-axis position data of the hand-held stylus was scaled such that there was a 1:1 gain relationship between movement on the tablet and movement of the object on the screen. In order to ensure a natural coupling, participants were made aware of the correspondence between their hand and the object movement, but no visual feedback was provided on the LCD screen. This should have helped participants focus attention on the object motion, as well as to minimise ongoing corrective movements that occur when vision on the hand and object are continuously available.

For the secondary task, participants were required to judge whether there was a change in the form or colour between successive (cue and probe) presentations of a stationary stimulus array. Four squares (each 0.25 deg) were initially presented for 500 ms (cue) on the LCD display, centred to the spatial and temporal midpoint of the disappearance of the moving object. After participants had given their response to the primary task, the four squares were presented again (probe) at a location coincident with the final position of the moving object (i.e., not the reappearance location). Participants were given 3000 ms to judge whether the squares had changed form or colour between the cue and probe presentation by pressing the z (no change) or the v (change) key of the computer keyboard with their left hand.

In the *Form* stimulus array, the four squares were each initially assigned an x and y location to coincide with the four corners of a larger square of 1 deg. Each of the four squares were then randomly shifted by −0.25, 0 or +0.25 deg. For the probe presentation, the four squares were either assigned the same location or all were randomly shifted again by −0.25, 0 or + 0.25 deg. For the *Colour* stimulus array, the four squares were each assigned an x and y location to coincide with the four corners of a larger square of 1 deg, and then randomly assigned a colour (red, magenta, blue and green) with no repetition. For the probe presentation, the four squares were either assigned the same colour or the colour of all four squares were randomly assigned a second time with no repetition. In the *Control* stimulus array, participants were informed that there would be no change between the cue and probe presentation of four back squares, which were each assigned an x and y location to coincide with the four corners of a larger square of 1 deg. Having given their response to the secondary change-detection task, participants were presented with a rest period, adjusted according to the occlusion duration, during which time the grey screen remained blank between 6000 and 6400 ms.

There were 6 unique combinations of Stimulus Array (*Control, Colour, Form*) and Tracking (*OC, OM*), which were presented in a randomised block order. In blocks with the *Control* stimulus array, participants performed 24 randomly-ordered trials, with 6 trials for each Position Step (−4, −2, +2, +4 deg). For blocks with the *Colour* or *Form* stimulus array, there was a randomly-ordered change between cue and probe in 12 of the 24 trials. Position Step was interleaved in these blocks, such that there was an equal distribution for trials with a change or no change in the stimulus array. For each trial, we evaluated the judgement accuracy of both the primary and secondary tasks, as well as the response time for the secondary task.

### Data acquisition and analysis

#### Ocular pursuit

Eye position (relative to display reference system) and eye velocity (relative to head reference system) signals were exported using the Eyelink parser software. In addition, the software identified and labelled saccades and blinks in the x-axis and -y-axis eye position data. The criteria for saccade identification were a velocity threshold of 30 deg/s, acceleration threshold of 8000 deg/s^2^, and a motion threshold of 0.15 deg. Using routines written in Matlab® (MATLAB 2020b, The MathWorks, USA), we then derived desaccaded smooth eye velocity. To this end, identified saccades and blinks in the eye velocity trace, plus 5 additional data points at the beginning and end of the saccade/blink trajectory, were replaced by linear interpolation. Saccades were generally of small amplitude and short duration, making linear interpolation a simple and adequate method of signal restoration^13^. The desaccaded eye velocity data were then filtered with a zero-phase, low-pass (20 Hz) auto-regressive filter. From the resulting smooth eye velocity, we calculated for each trial, the average over 6 frames (i.e., 24 ms) prior to occlusion (*T1*), 128–152 ms after occlusion (*T2*), and 228–252 ms after occlusion (*T3*).

#### fNIRS

**Figure 5:**
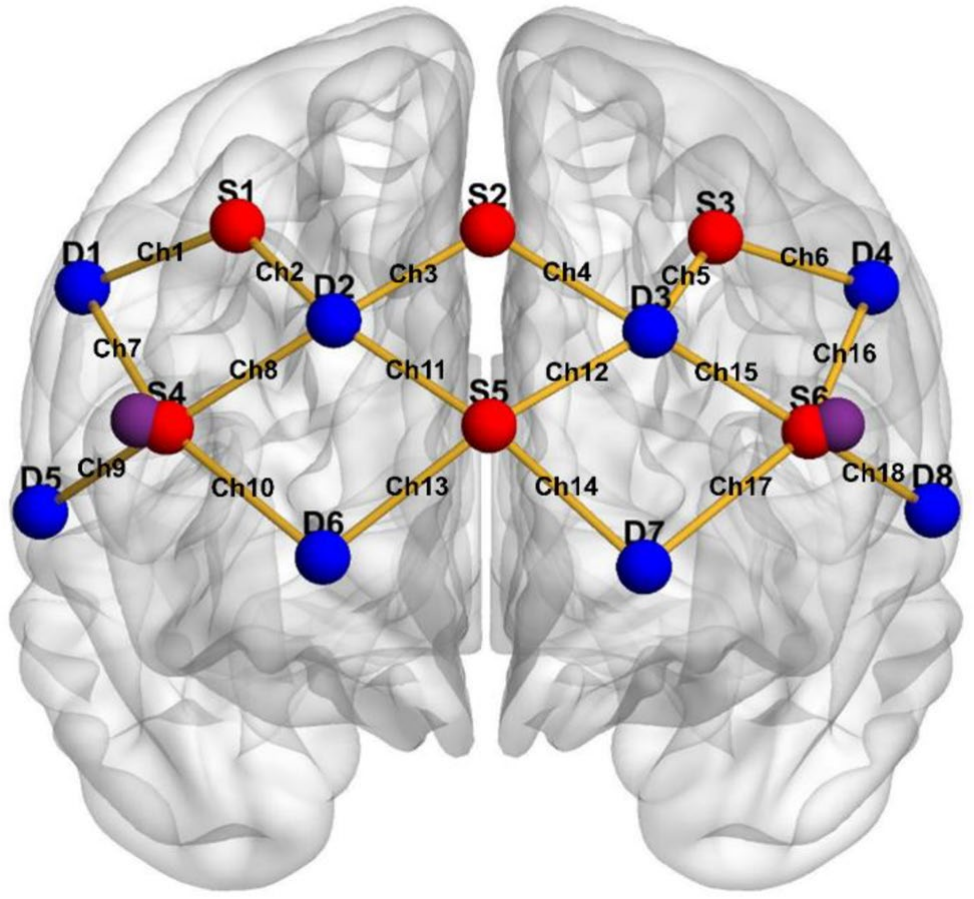
fNIRS optode organisation. The red circles represent sources, the blue circles represent detectors, and the purple circles represent short distance detectors. The channels are represented with yellow edges.

Relative change in oxy (O_2_Hb) and deoxy (HHb) haemoglobin while performing dual-task pursuit was quantified with a continuous wave NIRS system (BrainSight V2.3 system) that used two NIR wavelengths (705 and 830 nm) and a sampling rate of 10 Hz. The optodes (receivers and transmitters) were placed on the head of each participant using a cap (EasyCap) with holes cut at predetermined locations. The cap was placed by the same experienced researcher and was located relative to standard head landmarks (Nasion, Inion and Cz). A piece of black material was placed over the optodes to avoid potential crosstalk with ambient light from the room and IR light from the EyeLink illuminator. A 20-channel optode array (Fig. 5—NB. generated using BrainNet viewer toolbox^48^) corresponding to the links between 8 receivers and 6 transmitters, plus two proximity sensors, was used to cover the right and left PFC (4 dorsolateral channels and 5 medial channels for each hemisphere). Long-distance channels were positioned at around 3 cm, whereas the short distance channels were positioned at an inter-optode distance of around 0.8 cm, as recommended^49^. The Brodmann areas covered by the different channels were extracted via the NFRI function^50^ from the MNI (Montreal Neurological Institute) coordinates of the pre-cut cap holes.

Although fNIRS is a relatively resistant method to motion artifacts and is commonly used for quantification of brain activity during motor tasks, the fNIRS signal may still be affected when the participant moves their head, speaks, or when there is a momentary loss of contact between the scalp and the optodes. Noisy fNIRS signals can also result from the presence of too much light, which causes signal saturation (especially in prefrontal cortex because there is no hair). To minimize any unwanted impact of noise on the data analysis, for each participant, any channel not having a sufficient signal quality was excluded after observation of the power spectrum density of the O_2_Hb signals, where the presence of a cardiac rhythm in the signal (peak around 1 Hz) indicates good contact between the scalp and optodes^51^. Following this process, 2 participants were excluded for fNIRS analysis as it was deemed that too many channels had bad quality signal. An additional participant was removed (from all analyses) because they did not perform the task as instructed. Following the signal quality verification process, 9% of channels was removed for the remaining participants. Raw data (optical intensity) extracted from the BrainSight software (V2.3) was converted to optical density (OD, light absorption variation) using the Homer2 toolbox^52^. Next, two methods were used to reduce possible head motion artifacts. First, the moving standard deviation and spline interpolation method^53^ was applied using parameters: SDTresh = 20, AMPTresh = 0.5, tMotion = 0.5 s, tMask = 2 s and p = 0.99. Second, wavelet-based signal decomposition^54^ was used with parameter: iqr = 0.1, as recommended^55^. The OD time series were then converted into concentrations of O_2_Hb and HHb using the modified Beer-Lambert law^56^ corrected by a differential pathlength factor depending on the age of the participant^57^. A high (0.009 Hz) followed by a low pass (0.5 Hz) Butterworth zero phase digital filter (order 4) was applied to limit physiological artifacts. Finally, the short distance signal for each hemisphere was regressed to the long-distance channels located in the same hemisphere using the Matlab function *regress*. Time series of O_2_Hb and HHb were extracted for each trial and baseline corrected using the mean value calculated from the 6000 ms fixation time before the start of the trial. Relative changes of O_2_Hb and HHb were then calculated from the mean of each time series and used in our following analysis of PFC activity.

Graphs metrics (see below) were calculated from 18-by-18 partial correlation matrices computed on mins 5–9 of the whole time series for each of the 6 conditions. After detrending, partial Pearson correlations, which represent the association between two channels while controlling the effect of the other 16 channels^40,58^, were calculated from the O_2_Hb signal of each participant for all pairs of channels using Matlab function *partialcorr*. The resulting correlation matrices were subjected to Fisher z-transformation and all negative connections were set to zero. These weighted positive matrices were used to extract two measures of network efficiency: global efficiency, which is the average of inverse shortest path length and reflects the efficiency of information exchange in the whole network; local efficiency, which is the global efficiency computed on the neighbourhood of the node (i.e., channel) and reflects the information exchange between the immediate neighbour of a given node^40^. These efficiency metrics were calculated using functions implemented in the Brain Connectivity Toolbox^59^.

### Statistics

Judgment data from the primary and secondary tasks were analysed using generalized linear mixed modelling with a logit link function, whereas response time (secondary task), eye velocity and fNIRS data were analysed using linear mixed modelling (lme4 package v1.1–32 in RStudio 2023.03.0). Starting with the full fixed effects model in which each participant had a random intercept, an iterative, top-down process was followed in order to find the simplest model that best fit the data. Fixed effects were sequentially removed based on their statistical significance determined using Wald Chi Squared tests (CAR package v3.1–2), and those that returned p-values of 0.1 or less were provisionally retained. Model fit at each iteration was compared using conditional R^2^ (MUMIn package v1.47.5 for logistic models; piecewiseSEM v2.3.0 for linear models) and AIC, with final model selection based on the outcome of a Likelihood Ratio Test (anova in R). Having determined the final reduced model, fixed effects at p < 0.05 were further analysed using Bonferroni-corrected pairwise comparisons (EMMEANS package v1.7.2). To provide a measure of effect magnitude, odds ratio for generalised linear mixed models, and mean differences for linear mixed models, are presented.

### Supplementary Information

Supplementary Information.

## Data Availability

The datasets generated and analysed during the current study are available from the corresponding author on reasonable request.
