# Supplementary material for: Prefrontal cortex activity and functional organisation in dual-task ocular pursuit is affected by concurrent upper limb movement
Source: Sci Rep. 2024 May 1;14:9996. doi: 10.1038/s41598-024-57012-2 (PMC11063197; doi:10.1038/s41598-024-57012-2)
Supplement: Supplementary file 1 — Supplementary Information. [file 41598_2024_57012_MOESM1_ESM.docx]

Supplementary Material:

Bonferroni-corrected pairwise comparisons on main effect of Channel for local efficiency indicated the following differences:

Channel *1* lower (p < 0.01) than channel *3* (MD = 1.27e-02)*, 8* (MD = 1.29e-02)*, 11* (MD = 1.16e-02)*, 12* (MD = 1.20e-02)*, 13* (MD = 1.17e-02) and *14* (MD = 1.26e-02)

Channel *16* lower (p < 0.05) than channel *3* (MD = 1.77e-02)*, 4* (MD = 1.25e-02)*, 7* (MD = 1.04e-02), *8* (MD = 1.80e-02)*, 9* (MD = 9.88-03)*, 10* (MD = 1.41e-02)*, 11* (MD = 1.66e-02)*, 12* (MD = 1.70e-02)*, 13* (MD = 1.67e-02)*, 14* (MD = 1.77e-02)*, 15* (MD = 1.23e-02)*, 17* (MD = 1.04e-02).

Channel *6* lower (p < 0.05) than channel *2* (MD = 1.18e-02)*, 3* (MD = 2.02e-02)*, 4* (MD = 1.50e-02)*, 7* (MD = 1.30e-02)*, 8* (MD = 2.05e-02)*, 9* (MD = 1.24e-02)*, 10* (MD = 1.66e-02)*, 11* (MD = 1.91e-02)*, 12* (MD = 1.95e-02)*, 13* (MD = 1.93e-02)*, 14* (MD = 2.02e-02)*, 15* (MD = 1.48e-02)*, 17* (MD = 1.29e-02) and *18* (MD = 1.20e-02)

Channel *5* lower (p < 0.05) than channel *3* (MD = 1.15e-02)*, 8* (MD = 1.18e-02)*, 11* (MD = 1.04e-02)*, 12* (MD = 1.08e-02)*, 13* (MD = 1.05e-02) and *14* (MD = 1.15e-02).
